# *Calcaridorylaimus castaneae* sp. n. (Nematoda, Dorylaimidae) from Bulgaria with an identification key to the species of the genus

**DOI:** 10.3897/zookeys.410.6955

**Published:** 2014-05-20

**Authors:** Sevdan Nedelchev, Milka Elshishka, Stela Lazarova, Georgi Radoslavov, Peter Hristov, Vlada Peneva

**Affiliations:** 1 Faculty of Biology, University of Sofia, Bd. “Dragan Tzankov” 8, 1421 Sofia, Bulgaria; 2 Institute of Biodiversity and Ecosystem Research, Bulgarian Academy of Sciences, 2 Gagarin Street, 1113 Sofia, Bulgaria

**Keywords:** Taxonomy, morphology, 18S and D2-D3 rRNA genes, compendium

## Abstract

An unknown species belonging to the genus*Calcaridorylaimus* Andrássy, 1986 was collected from the litter of broadleaf forests dominated by *Castanea sativa* Mill. and mixed with *Quercus daleshampii* Ten. and *Fagus sylvatica* L. on Belasitsa Mountain, south-western Bulgaria. *Calcaridorylaimus castaneae* **sp. n.** is characterised by its long body (1.4–2.1 mm), lip region practically not offset, vulva transverse, short odontostyle (14.5–16 μm) and tail (75.5–110.5 μm, c=14.7–23.6; c’=2.9–4.4) in females and 38–46 μm long spicules with small spur before their distant end in males. It is most similar to *C. andrassyi* Ahmad & Shaheen, 2004, but differs in having transverse vs pore-like vulva and shorter spicules (38–46 μm vs 52–57 μm). An identification key to the species of the genus *Calcaridorylaimus* is proposed. Phylogenetic analyses were performed on 18S and D2-D3 expansion domains of 28S rRNA genes by Neighbor-Joining, Maximum Likelihood and Bayesian Inference methods. The phylograms inferred from 18S sequences showed closest relationships of the new species with some species belonging to the genus *Mesodorylaimus*. However, insufficient molecular data for members of both genera do not allow the phylogenetic relationships of *Calcaridorylaimus* and the new species described herein to be elucidated.

## Introduction

**Figure 1.**
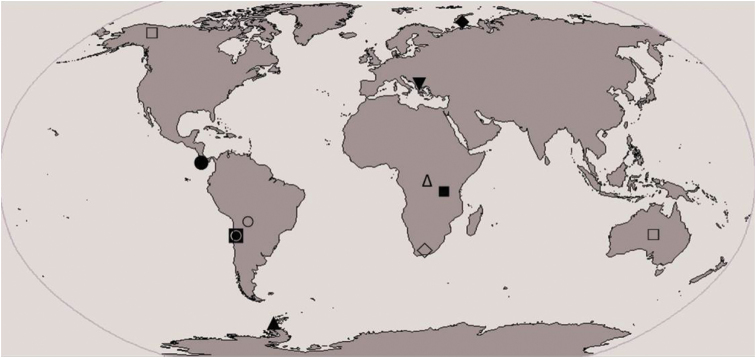
Distribution of *Calcaridorylaimus* spp. (■ *Calcaridorylaimus calcarifer*, ● *Calcaridorylaimus andrassyi*, ♦ *Calcaridorylaimus arcticus*, □ *Calcaridorylaimus promissus*, ▲ *Calcaridorylaimus signatus*, ○ *Calcaridorylaimus simillimus*, ♢ *Calcaridorylaimus sirgeli*, △ *Calcaridorylaimus ruwenzorii*, ◙ *Calcaridorylaimus beatus*, ▼ *Calcaridorylaimus castaneae* sp. n.).

During an ecological study of chestnut forests on Belasitsa Mountain (2003-2005) an undescribed species belonging to the genus *Calcaridorylaimus* Andrássy, 1986 was recovered. The genus *Calcaridorylaimus* is represented by nine species worldwide: *Calcaridorylaimus calcarifer* Andrássy, 1986, *Calcaridorylaimus promissus* Andrássy, 1986, *Calcaridorylaimus ruwenzorii* (De Coninck, 1935) Andrássy, 1986, *Calcaridorylaimus signatus* (Loof, 1975) Andrássy, 1986, *Calcaridorylaimus simillimus* Andrássy, 1986, *Calcaridorylaimus sirgeli* Heyns & Meyer, 1995, *Calcaridorylaimus arcticus* Gagarin, 1997, *Calcaridorylaimus andrassyi* Ahmad & Shaheen, 2004 and *Calcaridorylaimus beatus* Andrássy, 2011. The genus is distributed mainly in the southern hemisphere: three species occur in Africa, two in South America and one each in Antarctic, Central America, and Europe, and *Calcaridorylaimus promissus* was recorded from Australia and Alaska, North America (Andrássy 1986, 2003) (Fig. 1). The most characteristic features of these species are the shapes and structures of the spicules which are provided with a small spur before the distal tip. The new species is described based on both morphological and molecular data.

## Materials and methods

### Sample collection

The litter samples were collected in 2003 by the last author (VP) from three sites on Belasitsa Mountain representing different types of broadleaf forests dominated by *Castanea sativa* Mill. mixed with *Quercus daleshampii* Ten. and *Fagus sylvatica* L. (Forest Management Plan database, sub-compartments 104g, 140b and 146a). Subsequently, on 17.10.2012 new litter samples were collected by Dr Michaela Ilieva from one of these sites, sub-compartment 140b, in order to obtain fresh material for molecular studies. Nematodes were recovered from the litter using the Baermann funnel method. They were killed by heat (65 °C), fixed in TAF (Triethanolamine-formalin, Courtney et al. 1955), and processed to anhydrous glycerine (Seinhorst 1959). Drawings were prepared using an Amplival 30-G048b and a drawing tube РА-6У42. Photographs were taken using an Axio Imager M2-Carl Zeiss compound microscope equipped with a digital camera (ProgRes C7) and specialised software (CapturePro Software 2.8). Measurements were made using an Olympus BX41 light microscope, a digitising tablet (CalComp Drawing Board III, GTCO CalCom Peripherals, Scottsdale, AZ, USA), and computer programme Digitrak 1.0f (Philip Smith, Scottish Crop Research Institute, Dundee, UK).

### DNA extraction, amplification and sequencing

Genomic DNA was extracted from two female and two male worms using a standard nematode digestion protocol (Holterman et al. 2006). Two overlapping fragments of 18S rRNA genes (~1600 bp) were amplified from each specimen using primer sets 988F (5`-CTC AAA GAT TAA GCC ATG C-3`) and 1912R (5`-TTT ACG GTC AGA ACT AGG G-3)` for the first fragment, and 1813F (5`-CTG CGT GAG AGG TGA AAT-3`, 2646R 5`-GCT ACC TTG TTA CGA CTT TT-3`) for the second fragment (Holterman et al. 2006). The D2/D3 expansion segments of the 28S rRNA gene (~900 bp) were additionally amplified from all specimens using the primers D2A (5`-ACA AGT ACC GTG AGG GAA AGT TG-3`) and D3B (5`-TCG GAA GGA ACC AGC TAC TA-3`) (De Ley et al. 1999). Each PCR reaction was performed under the following conditions: initial denaturation 94 °C for 5 min; 40 cycles (denaturation 94 °C for 30 sec; primer annealing 50 °C for 30 sec; extension 72 °C for 1 min), and final extension 72 °C for 10 min. PCR products were visualized on 1% agarose gel with GreenSafe (NZYtech) under visible and UV light. Fragment size was determined using GeneRuler™ 100 bp Ladder Plus (Ferments, Thermo Scientific). The amplified products were sequenced by Eurofins MWG Operon.

### Sequence and phylogenetic analysis

The sequences of the new species have been deposited in GenBank with the accession numbers KF717497 and KF717498 for the 18S and the D2-D3 rRNA genes, respectively. A BLAST (Basic Local Alignment Search Tool) search at NCBI (National Center for Biotechnology Information) was performed using the obtained sequences as queries to confirm their nematode origin and to identify the most closely related nematode sequences. The sequences revealing a similarity up to 97% and 85% with nematodes from various Dorylaimida families were included in the phylogenetic analyses of 18S and D2-D3 regions, respectively (Griffiths et al. 2006; Holterman et al. 2006; Meldal et al. 2007; Lesaulnier et al. 2008; Pedram et al. 2010; Pedram et al. 2011; Álvarez-Ortega and Peña-Santiago 2012a; 2012b; Donn et al. 2012; Álvarez-Ortega et al. 2013). The Multiple Sequence Alignments (MSA) of both datasets were performed using the Clustal Omega tool (Sievers et al. 2011) via the EBI webserver: http://www.ebi.ac.uk/Tools/msa/clustalw2/. Subsequently, the MSAs were manually optimised and trimmed using MEGA 5 (Tamura et al. 2011). *Eudorylaimus* sp. (family Qudsianematidae) was used as an outgroup taxon for both 18S and D2-D3 rDNA sequence datasets (accession numbers AY284800 and AY593037, respectively; Holterman et al. 2008). The phylogenetic reconstructions of three datasets D2-D3, complete and partial 18S rDNA were performed using Neighbor Joining (NJ), Maximum Likelihood (ML) and the Bayesian Inference (BI) algorithms and implemented in MEGA 5.0 and MrBayes v3.2.1 (Huelsenbeck and Ronquist 2001, Tamura et al. 2011, Ronquist et al. 2012). The NJ phylogenetic inferences were performed under the following settings: Maximum Composite Likelihood method for computing evolutionary distances; Gamma distributed rates among sites, estimated values set up to 0.3429 (D2-D3) and 0.05 (18S rDNA); 2000 bootstrap replications. A total of 755 and 1593 positions in the final datasets were used for both analyses, respectively. General Time Reversible model (GTR) plus Gamma distribution rates (G) and 1000 bootstrap replications were used as ML analyses settings for all datasets. The Bayesian MCMC tree searches were conducted using MrBayes 3.2.1. Each analysis was run for 10, 000, 000 generations with a sample frequency of 1000 generations. The first 25% of the chains discarded as burning and the remaining 75% trees kept to summarise the tree topology, branch lengths, and posterior probabilities (PP) of branch support. The evolutionary models for nucleotide substitutions were set up as for ML analyses. Convergence diagnostic values were calculated every 1000 generations with a predefined stop value equal to 0.01. A single strict consensus tree was visualised using FigTree v1.4.0 graphical viewer (http://tree.bio.ed.ac.uk/software/figtree/). Posterior probabilities values of ≥0.80 (BI) and bootstrap values of ≥70 (NJ and ML) were considered as credible support values for nodes.

## Taxonomy

### Calcaridorylaimus castaneae sp. n.

http://zoobank.org/9BF1D302-1986-47C5-B0D8-2F5DC75BD6D2

http://species-id.net/wiki/Calcaridorylaimus_castaneae

**Figure 2.**
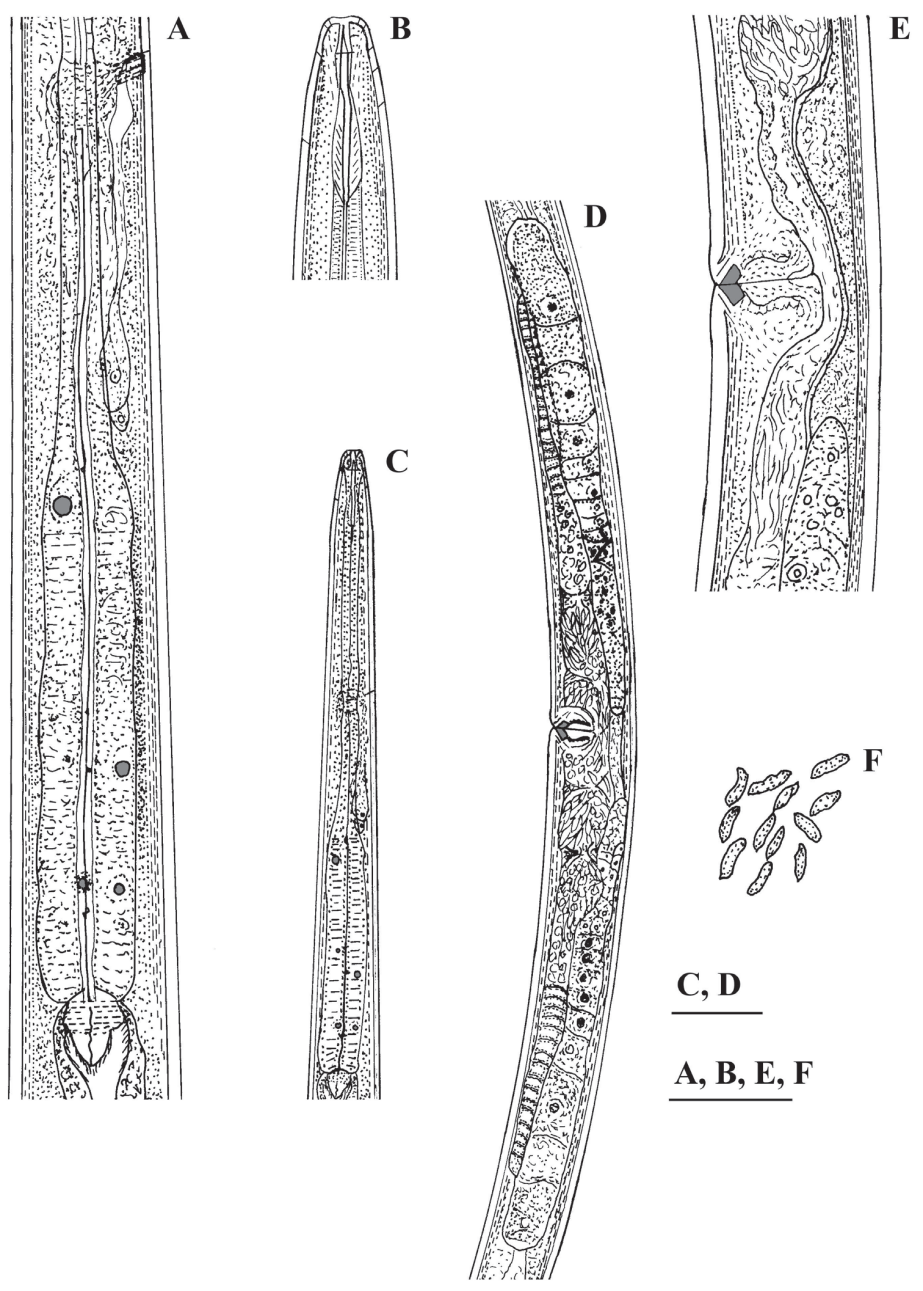
*Calcaridorylaimus castaneae* sp. n. *Female*: **A** Pharyngeal gland nuclei **B** Anterior region **C** Pharyngeal region **D** Genital system **E** Vulval region **F** Sperm cells in uterus. Scale bars: **A, B, E, F** – 30 μm; **C, D** – 50 μm.

Figs 2 [Fig F3] [Fig F4] [Fig F5] –6

#### Measurements.

See Table 1.

**Table 1.** Morphometrics of *Calcaridorylaimus castaneae* sp. n., females and males, from Belasitsa Mountain. All measurements, unless indicated otherwise, in μm and in the form: mean ± SD (range).

Habitat	Mixed broadleaf forest					Chestnut forest	
Locality and year of collection	140b 2003			140b 2012		104g 2003	
Characters	Holotype	Paratypes					
		Females	Males	Females	Males	Females	Males
n		40	20	10	10	18	12
L (mm)	1.7	1.7±0.11 (1.5–2.0)	1.6±0.08 (1.4–1.8)	1.6±0.2 (1.4–1.8)	1.6±0.1 (1.4–1.8)	1.6±0.15 (1.4–2.1)	1.5±0.06 (1.4–1.6)
a	35.7	36.6±2.4 (32.5–42.7)	37.9±2.3 (33.3–42.2)	35.7±2.1 (32.2–38.7)	40.0±2.3 (37.0–44.3)	38.4±1.4 (35.7–40.6)	38.1±2.0 (34.0–41.6)
b	4.9	5.0±0.3 (4.5–5.7)	4.7±0.2 (4.4–5.1)	5.1±0.2 (4.8–5.6)	5.1±0.4 (4.7–6.0)	4.9±0.3 (4.4–5.5)	4.6±0.2 (4.3–5.0)
c	19.4	18.8±0.4 (16.0–22.0)	74.4±7.4 (63.8–89.7)	18.3±1.4 (16.8–21.0)	66.7±6.9 (53.7–73.2)	18.5±2.3 (14.7–23.6)	72.2±7.0 (61.4–83.5)
c‘	3.9	3.6±0.4 (2.9–4.4)	0.76±0.06 (0.67–0.84)	3.5±0.4 (3.0–4.0)	0.84±0.06 (0.77–0.95)	3.6±0.3 (2.9–4.2)	0.76±0.06 (0.68–0.87)
V/T %	50.7	51.6±1.7 (47.9–54.8)	66.3±3.2 (60–72)	51.2±1.6 (49–54)	59.5±2.5 (56–62.5)	51.0±2.3 (46.5–54.5)	62.8±4.3 (54–70)
G1%	17	18.4±1.7 (14-22.6)	-	19.1±1.0 (18.1–20.7)	-	17.8±2.1 (13.8–21.7)	-
G2%	16	18.3±2.3 (14.1–24.4)	-	18.2±1.0 (16.6-19.1)	-	18.7±3 (14.5-26.5)	-
Odontostyle	14.5	15.0±0.4 (14.5–16)	15.1±0.25 (14.5–16)	15.1±0.2 (14.9–15.4)	14.9±0.4 (14–15)	15.0±0.2 (14.5–15)	15±0.3 (14.5–16)
Odontophore	26	24.8±2.2 (20–29)	22.9±1.5 (21–27)	21.6±2.1 (20–26)	23.7±4.0 (20–28)	27.2±1.6 (24–30)	25.6±1.7 (23–28)
Spear	41	39.8±2.2 (35–43)	37.7±1.2 (35.5–41.5)	36.8±2.0 (35–41)	38.6±4.2 (34–43)	41±1.4 (38–44)	40.7±1.6 (38–43)
Neck length	346	342±13.2 (310–366)	338.6±8.8 (318–352)	319.3±27.3 (272.5–352)	315±31.4 (242–340)	335.8±17 (306–376)	336±11.9 (310–352)
Cardia length	19	19±1.8 (16–24)	19±2.1 (16–24)	33.3±7.0 (22-41)	29.6±5.8 (24–39)	18.5±2.4 (15–25)	19.9±3.8 (14.5–26)
Body width at: lip region	12	12.3±0.3 (12–12.5)	12.3±0.3 (12–12.5)	12.4±0.5 (12–13)	12.5±0.5 (12–13)	12.3±0.4 (12–13)	12.5±0.19 (12–13)
mid-body	48	46.8±4.7 (39.5–55)	42.8±2.3 (37–47)	45.2±2.2 (41–48)	40.1±2.7 (35–44.5)	42±4.7 (37–58)	40.7±2.2 (37–45)
anus	22	24.9±1.8 (22–30)	28.8±0.9 (27–30)	25.7±1.1 (24.5–28.5)	28.7±1.7 (25–30.5)	24.8±1.3 (23–27)	28.5±1.0 (27–30)
Lateral chord	12	12.7±1.8 (10.5–18)	10.1±1.3 (8–12.5)	12.8±1.1 (11–15)	10.4±1.4 (9-13)	12.2±1.2 (10.5–16)	10±0.9 (8.5–12)
Prerectum	89	89.4±11 (68–117)	184±17.2 (132–207)	89.5±19.1 (64–104.5)	155.5±24.6 (130-200)	76.6±9.3 (60–96)	165.4±10 (145–176)
Rectum	41	37±2.4 (30–41)	41±2.1 (37.5–44)	34.5±4.2 (30–40.5)	40.3±3.3 (34-43)	39.6±1.4 (37–41)	43.8±1.5 (41–46)
Tail	88	90.9±7.1 (76–110)	22.1±1.8 (19–25)	88.7±8.5 (75.5–103)	24.3±2.6 (20–28)	90.1±9 (78–110.5)	21.6±2.3 (18–26)
Spicules	-	-	43±1 (41–45)	-	43.6±2.9 (38–46)	-	43.5±2 (39.5–45)
T-distance anterior end of anterior testis-cloaca (mm)	-	-	1.1±0.1 (0.9–1.3)	-	0.96±0.11 (0.86-1.11)	-	1.0±0.1 (0.8–1.15)

#### Description.

*Female.* Body slender, more or less curved ventrally. Cuticle ca 2 μm at anterior part of neck, 3 μm thick at midbody, 4.5–5.5 μm at postanal region; outer layer with fine transverse striae. Lateral chord ca 1/4 of body width. Three dorsal, two ventral and three lateral pores are observed in the spear area. Lip region practically not offset, lip region width ca 20% of its height. Lips partly fused, labial papilae slightly protruding. Body at proximal end of pharynx 3–4 times the width of the lip region diameter. Amphidial aperture 5–6 μm wide or about half the lip region width. Odontostyle 1.2–1.3 times the lip region diameter, aperture occupying 35–40% of its length. Odontophore simple 1.3–1.7 times odontostyle length. Guiding ring at 8.5–9 μm from anterior end. Nerve ring surrounding the pharynx at 36–39% of neck length from head end. Hemizonid and conspicuous excretory pore observed in the nerve ring region. Pharyngeal characters (five females and five males): pharynx beginning to widen at 56–60% and attaining its full width at 61–64% of neck length from anterior end. DO (for terminology see Loof and Coomans 1970) lying near the point where the pharynx attains its full width; DO–DN 8–13 μm. The two S_1_N lying at a small distance behind the middle of the distance DN–S_2_N, the anterior one (S_1_N_1_) smaller, ca 2 μm diam; S_1_N_2_ comparatively large and distinct, 3–4 μm diam. DN nucleolus 4–4.5 μm diam.; S_2_N 2–3 μm diam. (DN>S_1_N_2_>S_2_N). Locations (%):

**Table:** 

DO=60–64	S_1_N_1_=78-81	S_2_N=91–92	K=73–84
DN=62–66	S_1_N_2_=82–85	S_2_O=93–95	K’=78–85
DO–DN=2.2–3.8	S_1_N_1_–S_1_N_2_=2.9–4.8.		

Cardia conoid, variable in length, microvilli visible only here. Genital system didelphic-amphidelphic, ovaries reflexed, reaching rarely the vulva level. Oviducts and ovaries very long compared to uteri. Uteri not differentiated, short, anterior 96-125 μm and posterior 95-135 μm long, in one female with no sperm inside the genital system uteri shorter, 83 μm each, sphincter between uterus and *pars dilatata oviductus* well developed. Sperm present in uteri and *pars dilatata oviductus*. Synchronous uterine eggs 1–3, measuring 72–78 × 32–44 μm. Vulva transverse. Vagina extending to 60–70% inwards: *pars proximalis* 9–13 μm wide, 14.5–19.5 μm long, *pars refringens* more or less rounded trapezoid, 10–12.5 μm wide, 4–6 μm deep; *pars distalis* approx. 1.5 μm (terminology following De Ley et al. 1993). Peculiar tongue-like valve present at intestine-prerectum junction. Rectum 1.3–1.5, prerectum 3–4 times anal body width long, respectively. Tail first conoid, then more or less uniformly tapering to a narrowly rounded terminus. Posterior part of tail usually slightly curved dorsally. Two pairs of caudal pores, one subventral, other subdorsal.

*Male.* General morphology similar to that of female, body curved ventrally in J-shape when fixed. Genital system diorchic, testes opposed, well developed. Spicules dorylaimoid, with double contour on dorsal arm, 1.3–1.6 times the corresponding body diameter long; ventral arm smaller than dorsal. A spur present dorsally before the distal tip, distinctly visible in extruded spicules. Lateral guiding pieces 9-11 μm long or ca. 23 % spicule length. In addition to adcloacal pair seven to twelve (mostly nine or ten), regularly spaced ventromedian supplements present (9 supplements in 8 specimens; 10 suppl. – in 7, and 7, 8 and 12 each in one specimen). Prerectum 4.5–7.5 times the corresponding body diameter long, extending 0.7–1.8 body widths anterior to the supplement series. Tail dorsally conoid and broadly rounded. One subdorsal and one subterminal pair of caudal pores.

#### Differential diagnosis and relationships.

*Calcaridorylaimus castaneae* sp. n. differs from all species in the genus by a combination of the following characters: long body (1.4–2.1 mm), lip region practically not offset, short odontostyle (14.5–16 μm in females and 14-16 μm in males) and short female tail (75.5–110.5 μm, c=14.7–23.6; c’=2.9–4.4); vulva transverse, 38–46 μm long spicules with spur before its distal end. The new species is most similar to *Calcaridorylaimus andrassyi* from which it can be differentiated by having transverse vulva vs pore-like and shorter spicules (38–46 μm vs 52–57 μm). Further, *Calcaridorylaimus castaneae* differs from *Calcaridorylaimus ruwenzorii* by having shorter odontostyle (14.5-16 μm vs 19.5-25 μm), and tail (75.5-110.5 μm vs 160 μm, higher c (c=14.7-23.6 vs c=10) and lower c’ (c’=2.9–4.4 vs 7) values in females. It can be differentiated from *Calcaridorylaimus arcticus*, *Calcaridorylaimus beatus*, *Calcaridorylaimus calcarifer* and *Calcaridorylaimus signatus* by having different vulva shape (transverse vs longitudinal) and shorter spicules (38–46 μm vs 57–67 μm; 48–55 μm; 52–54 μm and 72 μm). From *Calcaridorylaimus sirgeli* it differs by having transverse vulva vs pore-like, higher c (c=14.7–23.6 vs c=9.7–11.3) and lower c’ (c’=2.9–4.4 vs c’=5.3–6.7) values. Finally, *Calcaridorylaimus castaneae* differs from *Calcaridorylaimus promissus* and *Calcaridorylaimus simillimus* by having longer odontostyle (14.5–16 μm vs 13 μm and 11 μm) and shorter tail (75.5–110.5 μm vs 158–178 μm and 175 μm).

Presence of the conspicuous excretory pore observed in *Calcaridorylaimus castaneae* is unusual for members of Dorylaimida, however, similar structure has been mentioned only for two *Longidorus* species, namely *Longidorus macrosoma* Hooper, 1961 and *Longidorus carniolensis* Širca et al., 2011 (Aboul-Eid 1969, Širca et al. 2011) and for several *Mesodorylaimus* species origination from Antarctica – *Mesodorylaimus chipevi* Nedelchev & Peneva, 2000, *Mesodorylaimus antarcticus* Nedelchev & Peneva, 2000, *Mesodorylaimus masleni* Nedelchev & Peneva, 2000, *Mesodorylaimus imperator* Loof, 1975 (Nedelchev and Peneva 2000).

#### Type locality and plant association.

Belasitsa Mountain, south-western Bulgaria, litter from old broadleaf forest (100-140 years) dominated by *Castanea sativa*, mixed with *Quercus daleshampii* and *Fagus sylvatica*. Site is located in the vicinity of Belasitsa hut, 41°22'12"N, 23°11'12"E (sub-compartment 140b). Second locality: young sweet chestnut forest (30-40 years old) near Belasitsa village (sub-compartment 104g).

#### Type material.

Holotype and 80 paratype females and 47 males deposited in the nematode collection of the Institute of Biodiversity and Ecosystem Research, Sofia, Bulgaria. Other paratypes deposited as follows: four females and two males in the Nematode Collection of the Foodland Environment Research Agency, Sand Hutton, UK (former Rothamsted Nematode Collection); three females and three males in the USDA Nematode Collection, Beltsville, Maryland, USA; two females and two males in the Riverside Nematode Collection, University of California, Riverside, USA; four females, and four males in the Wageningen Nematode Collection (WANECO), Wageningen, the Netherlands; four females and three males in the Nematode Collection of the Zoology Museum of the Ghent University, Belgium.

#### Etymology.

The scientific name is derived from the generic name of dominant tree species, the sweet chestnut tree (*Castanea*) in the forest where this nematode was found.

**Figure 3. F3:**
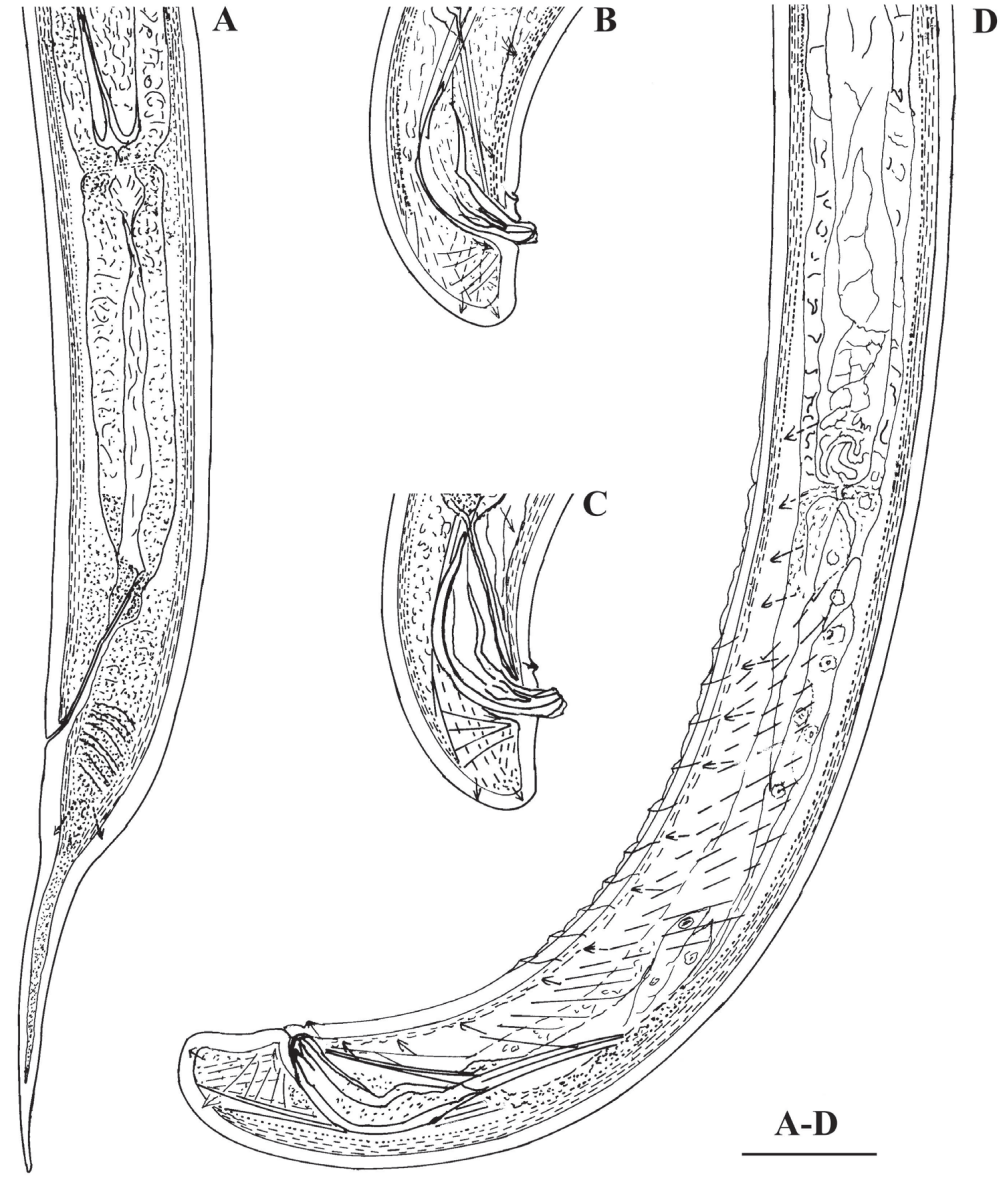
*Calcaridorylaimus castaneae* sp. n. *Female*: **A** Posterior region. *Male*: **B**, **C** Extruded spicules with supplements **D** Posterior region. Scale bar: **A–D** – 30 μm.

**Figure 4. F4:**
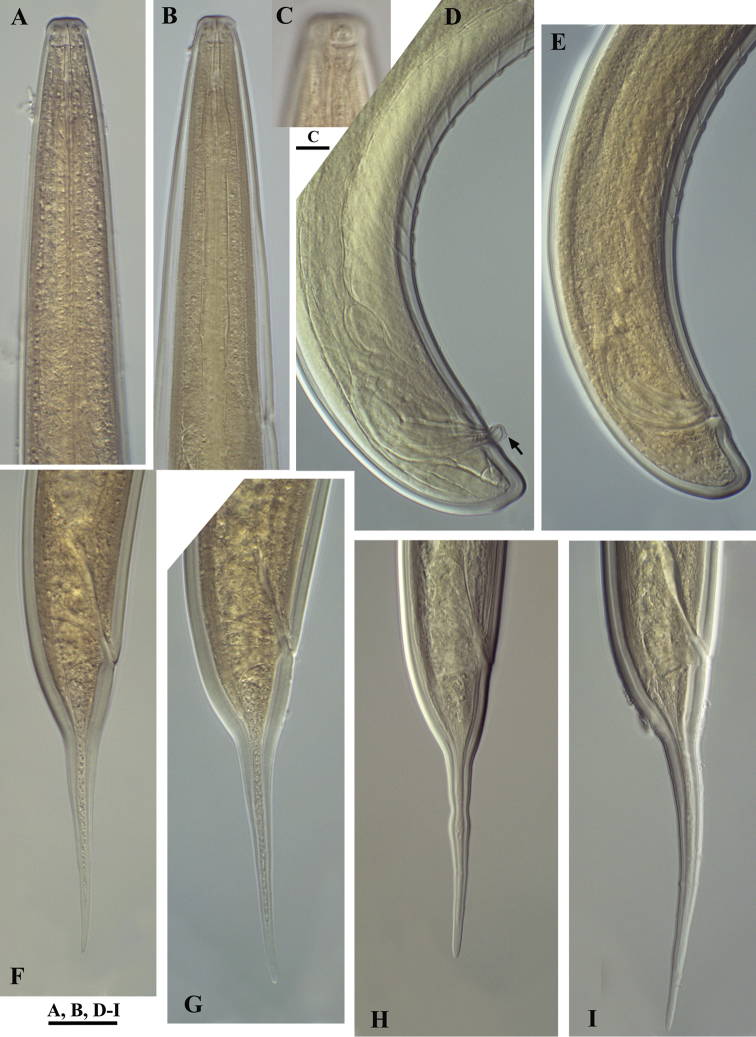
*Calcaridorylaimus castaneae* sp. n. *Female*: **A** Anterior end **C** Amphid **F–I** Tail shapes *Male* **B** Anterior end **D** Posterior end with extruded spicules, arrow indicating the spur **E** Posterior end. Scale bars: **A, B, D–I** – 20 μm; **C** – 6 μm.

**Figure 5. F5:**
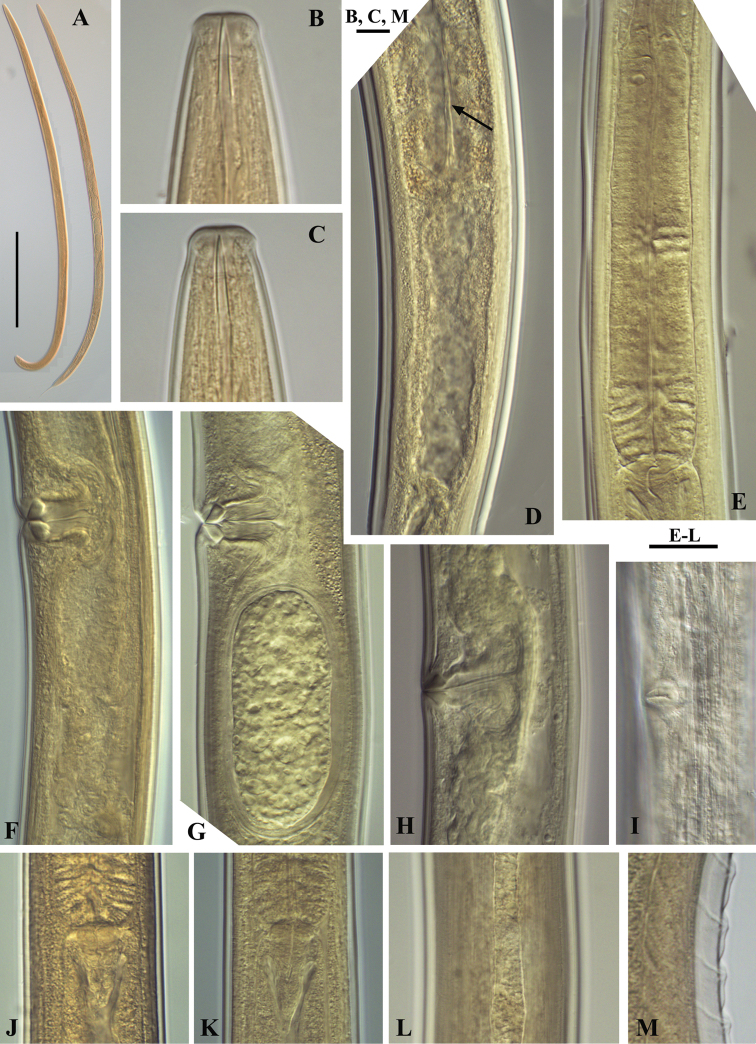
*Calcaridorylaimus castaneae* sp. n. *Female*: **A** Entire body **B** Lip region **D** Prerectum, arrow pointing tongue-like valve **E** Pharyngeal bulb **F** Vulval region with posterior uterus **G** Vulval region with egg in posterior uterus **H, I** Vulval region **J** Cardia **L** Lateral field. *Male*: **A** Entire body **C** Lip region **K** Cardia **M** Supplements. Scale bars: **A** – 200 μm; **B, C, M** – 6 μm; **E–L** – 20 μm.

**Figure 6.**
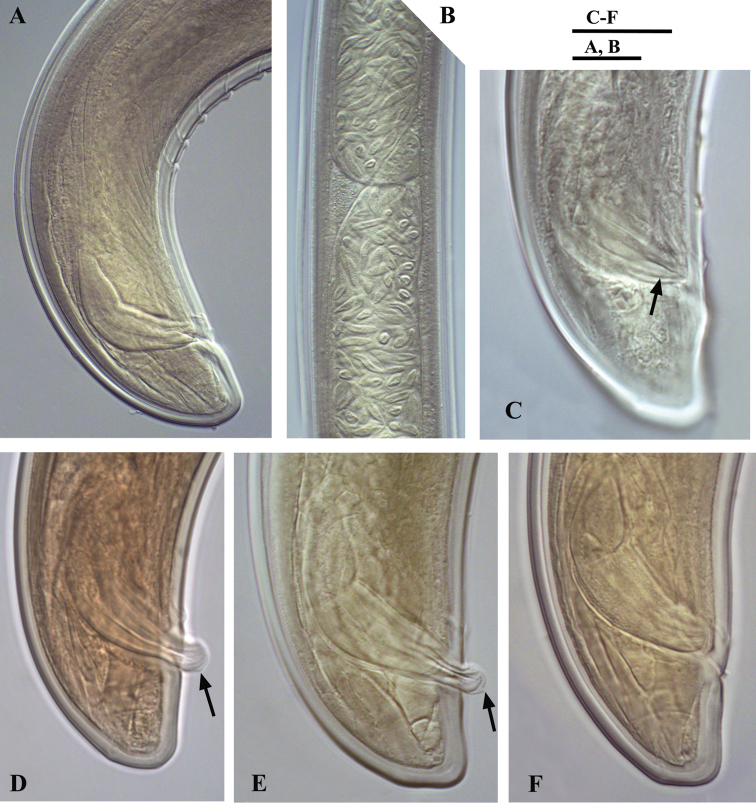
*Calcaridorylaimus castaneae* sp. n. *Male*: **A** Posterior end **B** Sperm cells in testis **C–F** Spicular region **C** Lateral piece of spicules **D, E** Extruded spicules, arrows pointing the spur **F** Spicules in the body. Scale bars: **A, B** – 20 μm; **C–F** – 18 μm.

### Key to species of *Calcaridorylaimus*

**Table:** 

1	Odontostyle 19.5–25 µm long	*Calcaridorylaimus ruwenzorii*
–	Odontostyle shorter, ≤17 µm	2
2	c’=11	*Calcaridorylaimus simillimus*
–	c’<9	3
3	c’=2.2–2.4	*Calcaridorylaimus beatus*
–	c’≥ 2.9	4
4	c≤11	5
–	c>11	7
5	V=45–47, supplements 10–13	*Calcaridorylaimus promissus*
–	V>49, supplements 6–9	6
6	Vulva longitudinal, spicules 52–54 µm	*Calcaridorylaimus calcarifer*
–	Vulva pore-like, spicules 39–45 µm	*Calcaridorylaimus sirgeli*
7	Lip region 14–17 µm wide, vulva longitudinal	8
–	Lip region 11–13 µm wide, vulva not longitudinal	9
8	Tail 120–171 µm long, L=1.6–2.27, spicules 57–67	*Calcaridorylaimus arcticus*
–	Tail 100 µm long, L=1.3–1.7, spicules 72	*Calcaridorylaimus signatus*
9	Vulva pore-like, spicules 52–57	*Calcaridorylaimus andrassyi*
–	Vulva transverse, spicules 38–46	*Calcaridorylaimus castaneae*

**Table 2.** Main morphological and morphometrical data of *Calcaridorylaimus* species, habitat type and distribution.

Species	Body length (mm)	a	c	c’	V%	Odontostyle	Lip region width	Tail	Vulva shape	Spicule length	Supple-ments	Habitat	Distribution	References
*Calcaridorylaimus andrassyi* ♀ ♂	1.8 (1.7–1.9) 1.5 (1.5–1.6)	44 (42–46) 39 (34–45)	15 (14–16) 78 (76–81)	4.4 (4.1–4.7) 0.66 (0.6–0.7)	49 (48–52)	15 (15–16) 15	12 (11.5–12) 12 (11.5–12.5)	113 (105–120) 20 (18.5–21.5)	P	54 (52–57)	10	forest	Costa Rica	Ahmad and Shaheen 2004
*Calcaridorylaimus arcticus*	1.6–2.3 1.84–2.24	24–34 24–34	13–16 53–75	3.5–5.8 0.6–0.7	49–53.4	14–17 14–17	15–17 15–17	139 (120–171) 36 (29–48)	L	63 (57–67)	10–12	aquatic, fresh-water	Russian Arctic	Gagarin 1997
*Calcaridorylaimus beatus*	1.28–1.30 1.36–1.48	24–29 28–33	19–23 57–72	2.2–2.4 0.6–0.8	51–52	15–16	14–15	60–68	L	48–55	13–14	Moist grassy soil, 4500 m asl	Chile	Andrássy 2011
*Calcaridorylaimus calcarifer*	1.18–1.30 0.95–1.07	30–32 26–28	7.8–10.3 45–46	6–8 0.7–0.8	49–52	13–14	10–12	115–167 21–23	L	52–54	8–9	rain forest soil	Republic of Congo	Andrássy 1986
*Calcaridorylaimus castaneae* sp. n.	1.4–2.1 1.4–1.8	32.2–42.7 33.3–44.3	16.0–23.6 53.7–89.7	2.9–4.4 0.7–0.95	46.5–54.8	14.5–16 14–16	12–13 12–13	75.5–110.5 18–28	T	38–46	7–12	broadleaf forest, litter	Bulgaria	Present study
*Calcaridorylaimus promissus*	1.28–1.37 1.02–1.10	36–38 28–30	7.7–8.4 47–57	8.4–9.0 0.8–0.9	45–47	13	10–11 -	158–178 18–22	T	50–53	10–13	wet moss from a rock, forest	Australia	Andrássy 1986
**Calcaridorylaimus promissus	1.6–1.65 1.55	34–37 35	9–11 90	7–8 ?	50–51	14–15	?-	150–160 ?	?	50	9–11	moss from swampy soil	Alaska	Andrássy 2003
*Calcaridorylaimus ruwenzorii*	1.6 1.3–1.5	32 35–40	10 44–74	7	47	19.5–25	12–14	160	?	44	7–8		Republic of Congo	de Coninck 1935
*Calcaridorylaimus signatus*	1.3–1.7 1.7	25–33 29	12.0–18.0 61	2.9–4.2 0.6*	49–56 -	16–18 17	14* -	100* 24*	L	72	12	wet moss	Antarctic	Loof 1975
*Calcaridorylaimus simillimus*	1.3 1.14	43 33	7.4 90	11 0.7	50	11.5	9.5–10	175 12	T	45	11	*Stipa* sp., near lake Titikaka	Bolivia	Andrássy 1986
*Calcaridorylaimus sirgeli*	1.34 (1.1–1.5) 1.36 (1.2–1.5)	33.2 (30–36) 37.7 (35–38)	10.6 (9.7–11.3) 77 (63–85)	6.1 (5.3–6.7) 0.7 (0.6-.8)	53.4 (52–56)	13.6 (12–15) 13.1 (12.5–14)	11.5 (10–12.5) 11.3 (10–12.5)	127 (105–148) 17.9 (15–22)	P	42.4 (39–45)	6–9	moist soil under fynbos and mosses	South Africa	Heyns and Meyer 1995

Shape of vulva: **L** – longitudinal; **P** – pore like; **T** – transverse; when average values are present ranges in parentheses; * from the drawing; ** *Calcaridorylaimus promissus* from Alaska (Andrássy 2003) was not included in a subsequent paper by the same author (Andrássy 2011); since most of the characters deviate substantially from the original description, probably this population belongs to another species.

### Phylogenetic relationships of *Calcaridorylaimus castaneae* based on partial 28S and 18S rDNA sequences

The sequences of D2-D3 expansion domains of 28S and 18S ribosomal DNA from two female and two male nematodes have been processed. No inter-individual variability within both domains have been observed, thus only two consensus sequences for each of the genes (787 and 1699 bp long) have been submitted to GenBank (accession numbers KF717498 and KF717497). A BLAST search for D2-D3 region showed highest similarity (88%) to the sequence of *Labronema vulvapapillatum* clone 2 (AY592997, Holterman et al. 2008) while the 18S rDNA showed 99% similarity (4-6 nucleotide differences) to the sequences of *Mesodorylaimus bastiani* (AJ966488) from France (Meldal et al. 2007) and two sequences of unidentified species from environmental samples from Scotland (AJ875133 and JN049666) (Griffiths et al. 2006, Donn et al. 2012).

**Figure 7.**
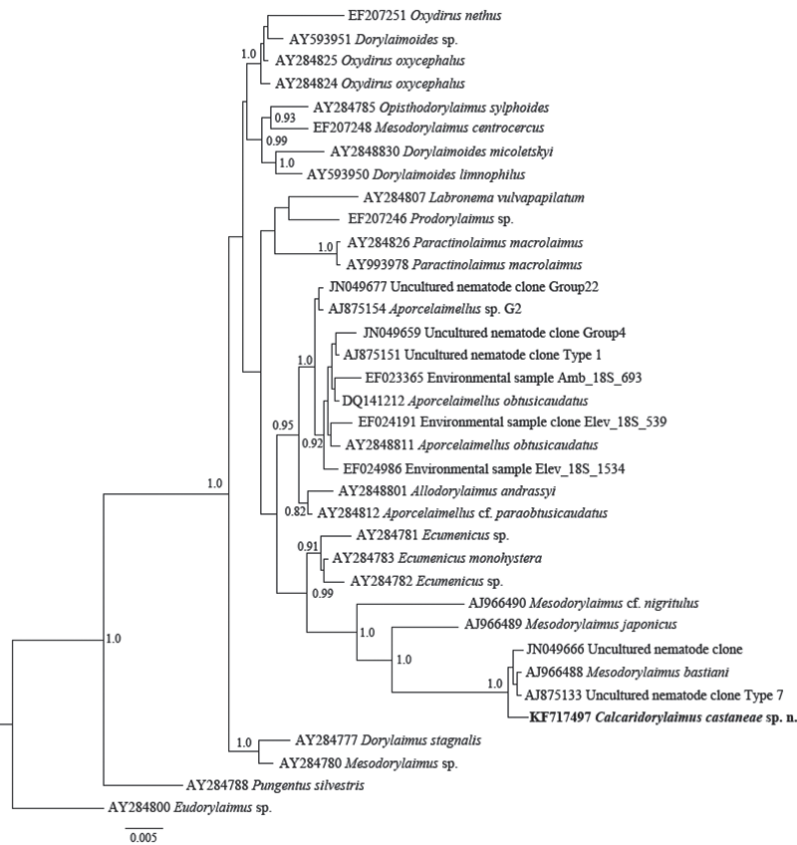
Phylogenetic relationships of *Calcaridorylaimus castaneae* sp. n. and its closest species for 18S rRNA gene. Bayesian Inference strict consensus tree acquired under GTR+G model. Posterior probabilities higher than 0.8 are presented.

**Figure 8.**
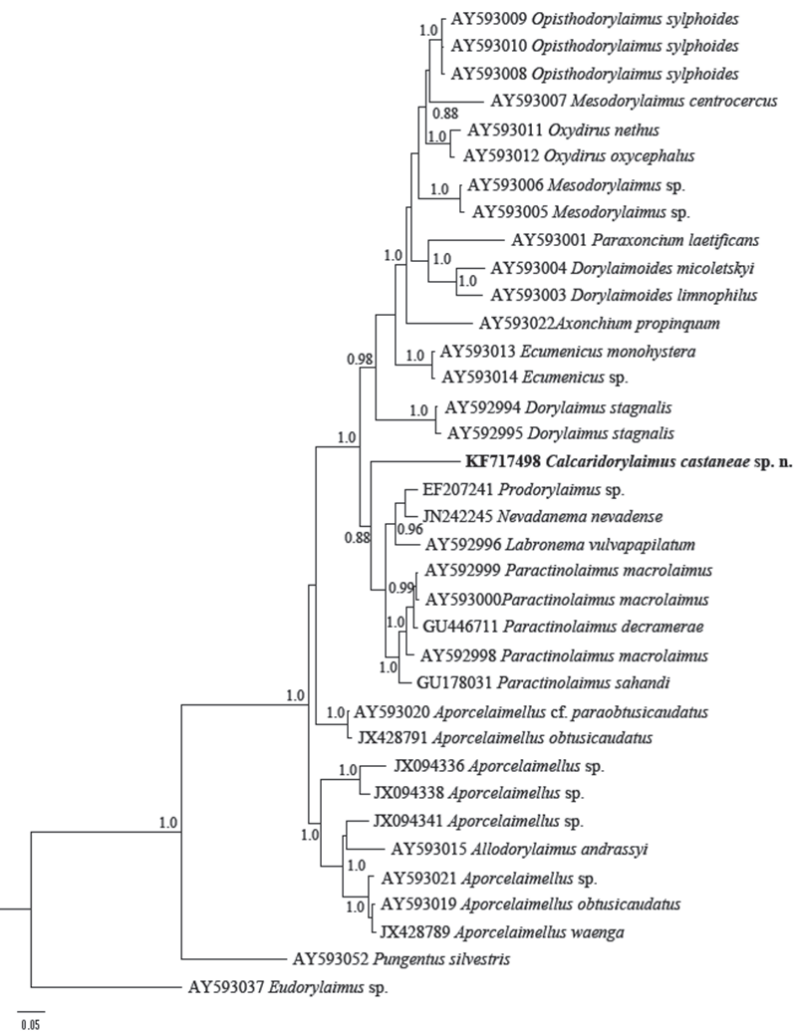
Phylogenetic relationships of *Calcaridorylaimus castaneae* sp. n. and its closest species for D2-D3 expansion segments of the 28S rRNA gene. Bayesian Inference strict consensus tree acquired under GTR+G model. Posterior probabilities higher than 0.8 are presented.

**Figure 9.**
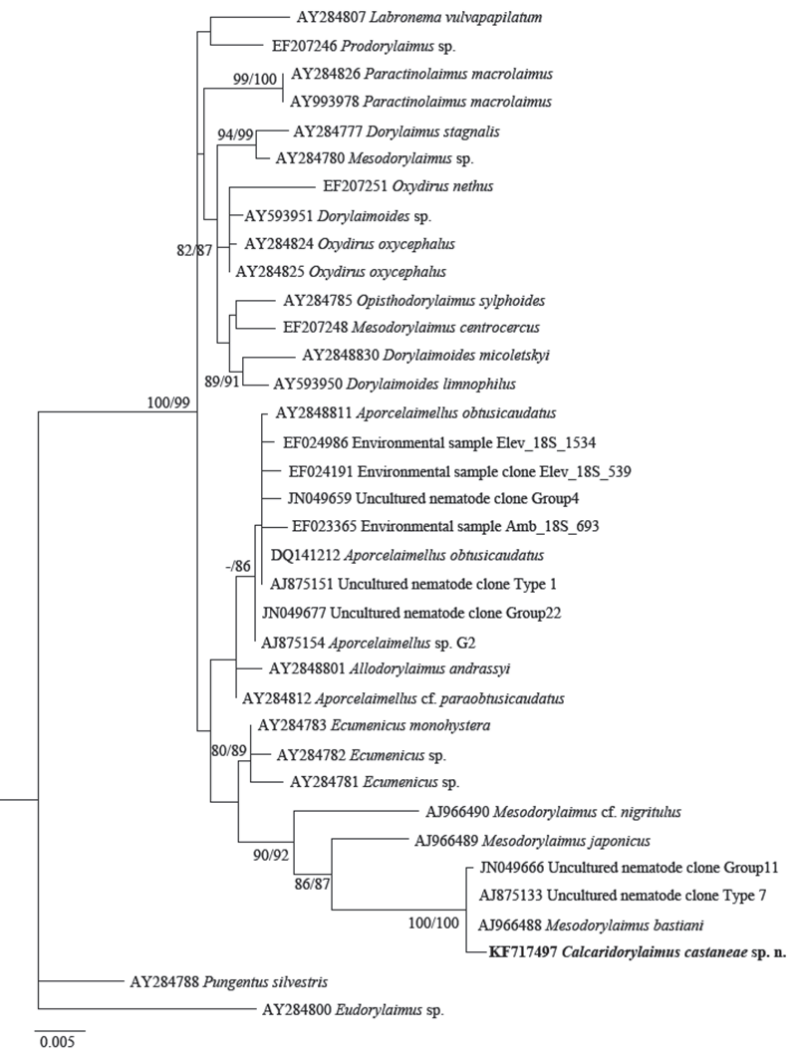
Phylogenetic relationships of *Calcaridorylaimus castaneae* sp. n. and its closest species for 18S rRNA gene. Tree acquired with Neighbor Joining and Maximum Likelihood (GTR+G model) methods. Bootstrap values higher than 70% are presented.

**Figure 10.**
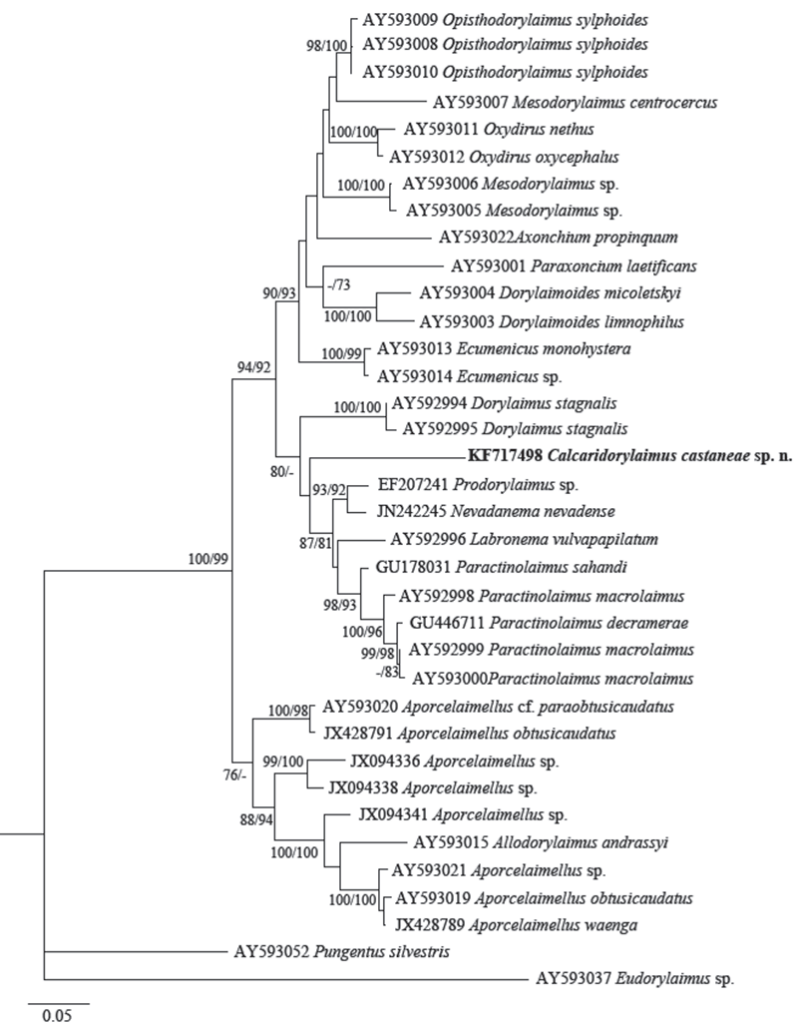
Phylogenetic relationships of *Calcaridorylaimus castaneae* sp. n. and its closest species for D2-D3 expansion segments of the 28S rRNA gene. Tree acquired with Neighbor Joining and Maximum Likelihood (GTR+G model) methods. Bootstrap values higher than 70% are presented.

The phylogenetic analyses based on 18S rDNA and D2-D3 of 28S rDNA sequences from various dorylaimid species with the highest matches of the BLAST search (up to 97% and 85%, respectively) were aligned along with our sequence. The phylograms obtained by NJ, ML and BI methods showed similar topology and differed only in the positions of poorly supported clades. The BI trees (Figs 7 and 8) with posterior probabilities higher than 0.8 and NJ-ML trees with bootstrap values above 70% are presented (Figs 9 and 10). The new species has clustered in a well-supported group with several *Mesodorylaimus* spp. (*Mesodorylaimus bastiani* (Bütschli, 1873), *Mesodorylaimus japonicus* (Cobb in Thorne and Swanger, 1936) and *Mesodorylaimus* cf. *nigritulus* (Schneider, 1937)) and two unidentified nematodes from environmental samples in the 18S rDNA phylogenetic reconstructions (Figs 7 and 9); and to more distantly related species from various dorylaimid genera and families (*Prodorylaimus*, *Labronema*, *Nevadanema*, *Paractinolaimus*) in the partial D2-D3 LSU reconstructions (Figs 8 and 10).

The genus *Calcaridorylaimus* was erected by Andrássy (1986) to accommodate a few species having different shapes and structures of spicules from those of *Mesodorylaimus*, with the males and females being practically indistinguishable. The phylograms inferred from 18S sequences showed the closest relationships of *Calcaridorylaimus castaneae* with some members of the latter genus; however, the insufficiency of molecular data complementary to detailed morphological studies of species belonging to both genera does not allow the elucidation of evolutionary relationships among them and the position of the new species herein described.

## Supplementary Material

XML Treatment for Calcaridorylaimus castaneae
